# Highlights of the 2025 American Joint Replacement Registry Annual Report

**DOI:** 10.1016/j.artd.2026.102121

**Published:** 2026-08-22

**Authors:** David Novikov, Niall Cochrane, James I. Huddleston, Brett R. Levine, Anna Cohen-Rosenblum

**Affiliations:** a Department of Orthopedic Surgery, New York University Grossman School of Medicine, New York, NY, USA; b Department of Orthopedic Surgery, Mayo Clinic, Jacksonville, FL, USA; c Department of Orthopedic Surgery, Stanford University, Stanford, CA, USA; d MedStar Orthopedic Institute, Georgetown University School of Medicine, Washington, DC, USA

**Keywords:** American Joint Replacement Registry, Outcomes, Total hip replacement, Total knee replacement, Trends

## Abstract

The 2025 American Joint Replacement Registry Annual Report includes 4.6 million hip and knee arthroplasty procedures, with 4,434,668 validated cases, representing a 20% increase from the prior report. Registry participation continues to expand, with contributions from over 5000 surgeons and 1200 institutions, including an increasing submission of patient-reported outcome measures. A key methodological change includes the adoption of Kaplan–Meier survivorship analysis, allowing improved assessment of time-dependent revision risk. Ongoing efforts to improve data completeness and registry infrastructure continue to support registry-driven research. The full report can be accessed at https://www.aaos.org/registries/publications/ajrr-annual-report/.

## American Joint Replacement Registry 2025 executive summary

The 2025 American Joint Replacement Registry (AJRR) Annual Report marks the 12th iteration of the report, capturing 4.6 million recorded primary and revision hip and knee arthroplasties, a 9% increase from approximately 4.3 million procedures captured in last year’s edition. Notably, the number of validated procedures, after excluding invalid data, increased 20% from 3,715,320 in the 2024 Annual Report to 4,434,668 in the 2025 Annual Report. More than 750 institutions are now actively submitting patient-reported outcome measures (PROM) data. This was among 5000 surgeons and 1200 contracted institutions, 960 of which submitted data, spanning all 50 states, with the highest case volumes reported in California, New York, and Florida.

### Updated survival analysis method

The methodology used to generate the cumulative percent revision curves was changed for the 2025 Annual Report. Previous annual reports generated these survivorship curves using the Cox proportional hazards methodology. For the 2025 Annual Report, survivorship was analyzed using the Kaplan–Meier (KM) method. Unlike Cox proportional hazard methodology, KM methodology does not assume that revision risk remains constant over time and thus provides a more accurate depiction of how survivorship and revision risk evolves throughout follow-up. Although the KM curves are not covariate-adjusted, the hazard ratios (HRs) below the figures are adjusted for age, sex, and Charlson Comorbidity Index. If the relative risk between groups was consistent over time, 1 HR was reported; if that risk changed throughout follow-up, separate HRs were reported for different time periods. This methodology is consistent with reporting standards adopted by other national registries [1]. Patients were censored at the end of the follow-up period, when a revision arthroplasty or death occurred.

### Bearing materials

Prior to the adoption of highly cross-linked polyethylene (HXPE), conventional polyethylene (CPE) wear, and osteolysis were one of the leading causes of hip and knee arthroplasty failure [2,3]. The 2025 Annual Report includes a comparative analysis of HXPE use in primary total hip arthroplasty (THA) in the United States (US) vs international registries. In addition, survival curves were generated to compare antioxidant HXPE compared to HXPE without antioxidant. Conventional polyethylene was not included as a comparator given its contemporary utilization of <1% of primary THA cases in the AJRR.

### Patient-reported o utcome measures

In 2024, the Centers for Medicare & Medicaid Services implemented mandatory reporting of Patient-Reported Outcome Performance Measures for patients aged 65 and older through the Hospital Inpatient Quality Reporting program [4]. Accordingly, the AJRR has updated its submission infrastructure to align with these requirements. The 2025 Annual Report includes an expanded PROMs section detailing completeness rates for several required Inpatient Quality Reporting variables. It also reports on the percentage of cases achieving thresholds for substantial clinical benefit and Patient Acceptable Symptomatic State for the Knee Injury and Osteoarthritis Outcome Score for Joint Replacement and the Hip Disability and Osteoarthritis Outcome Score for Joint Replacement, along with the percentage of cases that have achieved anchor-based and distributions-based minimally clinically important differences.

### Technology assistance

As technology use during primary hip and knee arthroplasty continues to grow in the US, the 2025 Annual Report includes a more detailed analysis of robotic and navigation use. In particular, overall growth trends as well as manufacturer-specific data are now available. Considering technology continues to shape the adult reconstruction landscape, efforts are under way to help standardize data completeness in the AJRR as well as across registries worldwide.

### Minimum datasets

**Figure 1:**
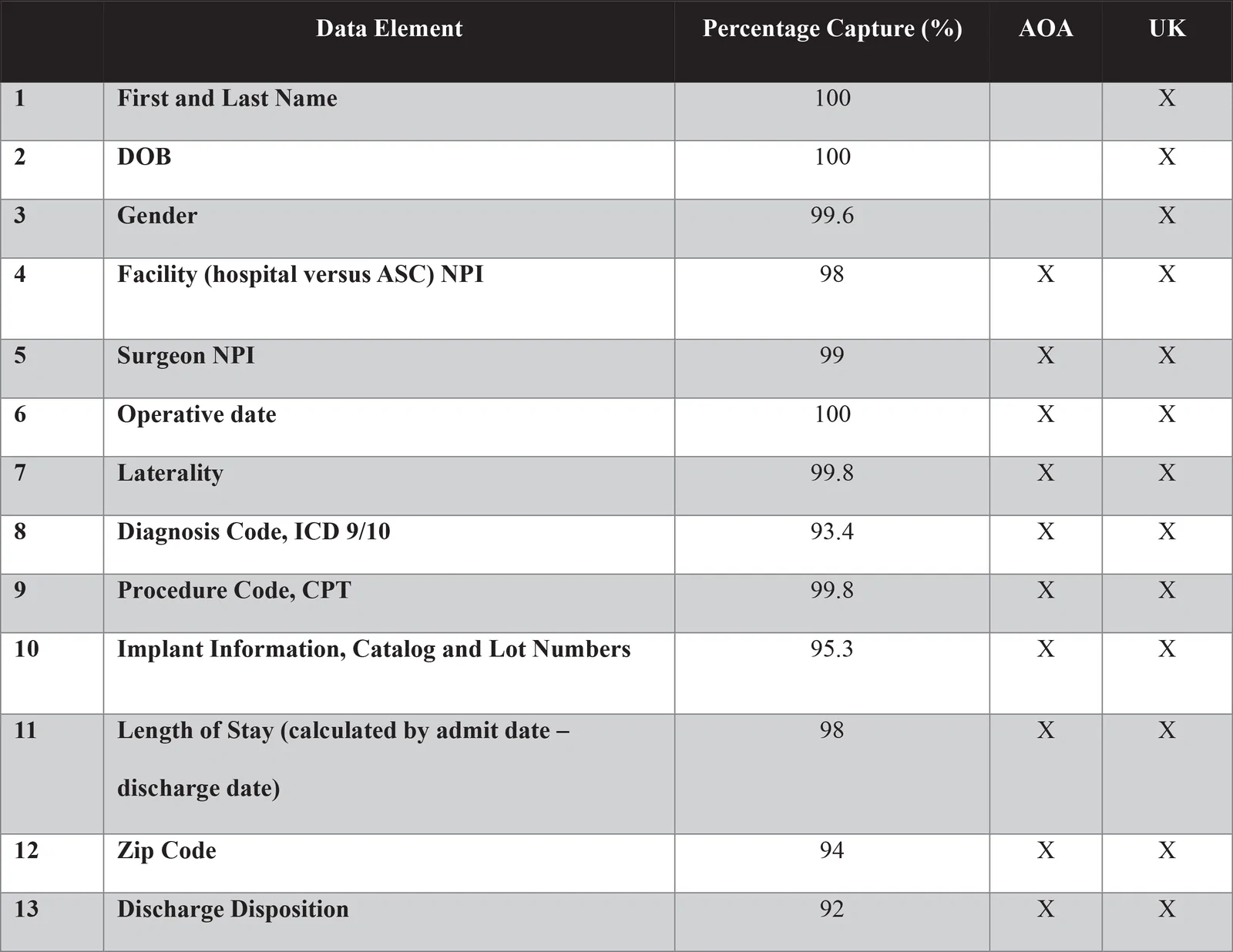
Level 1 minimum dataset.

In an effort to enhance data completeness and overall data quality, a minimum dataset has been created. The registry will no longer accept site data if all 17, level 1 data elements are not included (Fig. 1). A dashboard has been created to provide each participating site real-time visibility of the data elements that are required as well as where the deficiencies are. These measures can help sites meet the minimum dataset requirements to ensure data completeness.

### Registry analytics institute

The American Academy of Orthopaedic Surgeons Registry Analytics Institute helps advance the field of joint replacement by providing physicians and clinician-scientists AJRR data analyses to help answer research questions. There has been a major increase in requests with over 150 submissions this past year. This has led to significant scientific productivity with the publication of 20 peer-reviewed manuscripts and over 25 posters and presentations since the last report. Topics of interest included cementless total knee arthroplasty (TKA) and THA [5,6,7], periprosthetic joint infection [8], technology [9,10], patella resurfacing [11,12], and obesity [[13], [14], [15]].

## 2025 AJRR Annual Report highlights

The 2025 AJRR Annual Report captured 4,434,668 validated hip and knee procedures performed between 2012 and 2025 for analysis, representing an approximately 20% increase from the 3,715,320 validated procedures reported last year. The majority of procedures within the AJRR continue to be primary TKA (51%), followed by primary THA (32%). The cohort TKA and THA patients was predominantly female (59%) and identified as non-Hispanic White (89%), with race data unavailable for 16% of patients. The number of enrolled Ambulatory Surgery Centers continues to grow, increasing from 93 in 2023 to 107 contracted in 2024. The annual procedure volume from Ambulatory Surgery Centers has remained stable at approximately 18,000 cases over the last 3 years. The mean age for elective primary THA was 66 years and elective primary TKA was 68 years, consistent with prior annual reports. The mean age for partial knee arthroplasty was 65 years. The mean annual number of procedures per submitting surgeon increased from 44 in 2023 to 49 in 2024 for THA and from 65 in 2023 to 74 in 2024 for TKA.

### Trends consistent with 2024 AJRR report

• Postoperative length of stay for elective THA (1 day) and TKA (1 day) has remained constant since 2023 and is 1 day shorter than elective TKA (2 days) and elective THA (2 days) in 2018. This is likely the result of a higher incidence of same day discharge and outpatient THA and TKA.
• In patients with a femoral neck fracture (FNF), hemiarthroplasty (HA) remains the most common form of treatment at 72% in 2024 compared to 72% in 2023. Although the rates of THA for FNF have increased from 17% in 2012 to 28% in 2024, over the last 3 years the rates have been similar (28% in 2023, and 27% in 2022).
• Use of cemented fixation for femoral stems in THA for FNF remained constant at 24% after increasing from 13% in 2017 to 24% in 2023. Cemented fixation was more often used in HA (42%) compared to THA (24%) when treating FNF.
• Dual-mobility (DM) use in primary elective THA initially saw a rise in utilization from 4% in 2012 to 9% in 2020. Since that time, DM usage has remained constant (7% in 2025, 7% in 2023, and 7% in 2022).
• The preferred method of anesthesia continues to be spinal anesthesia in primary THA (47%) and primary TKA (37%), with a continued decline in general anesthesia (GA) use in isolation. In 2024, GA alone was used in 36% of primary THA cases (37% in 2023, and 38% in 2022) and in 22% of primary TKA cases (22% in 2023, and 25% in 2022).
• The rate of robotic assistance in primary TKA has significantly increased in the last few years with 16% of primary TKA cases that use it in 2024 compared to 9% in 2021. The use of navigation alone has remained low at 4% compared to 5% in 2023.
• The use of peripheral nerve blocks (PNBs) to supplement GA or spinal anesthesia have roughly been the same in 2024 compared to 2023 for both primary THA and TKA. In THA, GA + PNB declined from 4% to 3% of cases and spinal + PNB stayed stable at 3%. In TKA, GA + PNB declined from 15% to 14% and spinal + PNB stayed stable at 16%.

### Trends different from 2024 AJRR report

• In the oldest group of patients (>90 years old), cement for femoral fixation in HA is used 56% of the time, an increase from 54% in 2023 and 52% in 2022.
• In contrast to primary TKA, robotic assistance in primary THA decreased slightly in 2024, from 7% in 2023 to 6%. Similarly, the use of computer navigation alone in primary THA declined to 3% in 2024, compared to 5% between 2021 and 2023.

### Trends in primary THA

• The use of large femoral heads (≥36 mm diameter) in elective THA continues to steadily increase and is more popular than ever. The most commonly implanted size remains 36 mm and is used in 64% of cases in 2024, up from 63% in 2022. Larger heads (≥40 mm) have seen the highest use since the inception of the registry and continue to slowly increase in use as they were implanted in 13% of primary THA cases in 2024 compared to 11% in 2023 and 9% in 2022. Although 40 mm heads were associated with a higher cumulative rate of revision than 36 mm heads at both 0-2 years (adjusted HR 1.1, 95% confidence interval [95% CI] 1-1.08; *P* = .05) and 2-10 years (adjusted HR 1.4, 95% CI 1.2-1.5; *P* < .01), this analysis did not account for the potential use of 40 mm heads in patients at higher risk for instability. Further, AJRR is currently unable to accurately capture revisions for mechanically assisted crevice corrosion.
• Ceramic femoral heads remain the most frequently used femoral head material in primary THA at 83% of cases compared to 82% in 2023. Ceramic on polyethylene remains the most commonly used bearing surface in elective THA at 74% in 2024 up from 73% in 2023. Metal on polyethylene usage continues to decline, seeing its lowest use at 3% down from 4% in 2023. Similar to the 2024 Annual Report, metal on polyethylene has a significantly higher cumulative rate of revision at 1-10 years than ceramic on polyethylene (adjusted HR 1.3, 95% CI 1.26-1.44; *P* < .01) in Medicare patients 65 years of age and older.
• Following an increase to 5% in the 2024 Annual Report, the rate of cemented femoral fixation in elective primary THA remained constant at 5% in 2024. This remains lower than that reported by other international registries. For example, registry data from the Australia (35.7%), United Kingdom (42.5%), Canada (63.2%), and New Zealand (44.8%) registries indicates a considerably larger percentage of primary elective THA that are done with cement as the primary mode of femoral fixation [[16], [17], [18], [19]]. Cementless femoral fixation continues to be the predominant technique across all age groups, including patients older than 90 years, in whom it was used in 67% of cases. In women 65 years of age and older, cemented femoral fixation had a lower cumulative revision rate within the first 6 months after primary THA than cementless fixation (adjusted HR 0.6, 95% CI 0.5-0.7; *P* < .01).
• New in the 2025 Annual Report is the inclusion of an analysis of HXPE use in primary THA, particularly antioxidant HXPE and HXPE without antioxidant liners. Since the first AJRR report in 2012, antioxidant HXPE initially saw an increase in use from 7% in 2012 to a peak of 15% in 2019 after which it has steadily declined to 3% in 2024. Similarly, HXPE without antioxidant was used in 93% of cases in 2012, declined to 85% in 2019, and has steadily increased to 97% in 2024. In females <65 years, HXPE without antioxidant carried a lower revision risk within the first 2 years (adjusted HR 0.77; 95% CI 0.69- 0.87, *P* < .01) than antioxidant HXPE, with no significant difference thereafter (adjusted HR 1.08, 95% CI 0.93-1.3, *P* = .31). In females ≥65 years, the same pattern held through the first year (adjusted HR 0.78, 95% CI 0.7-0.87, *P* < .01), with no difference thereafter (adjusted HR 1.0, 95% CI 0.89-1.1, *P* = .89). In males, HXPE without antioxidant had a lower revision risk within the first year than antioxidant HXPE (<65 years: adjusted HR 0.64, 95% CI 0.55-0.73, *P* < .01; ≥65 years: adjusted HR 0.74, 95% CI 0.65-0.85, *P* < .01), before carrying a higher revision risk after 1 year regardless of age (<65 years: adjusted HR 1.35, 95% CI 1.14-1.6; *P* < .01; ≥65 years: adjusted HR 1.29, 95% CI 1.08-1.54; *P* < .01).

### Trends in revision THA

• Periprosthetic joint infection remained the leading cause of revision THA, comprising 26% of all hip revisions, similar to 26% in 2023. Instability remained the second leading indication for revision THA, accounting for 21% of all revision THA cases, up slightly from 20% in 2023.
• Dual-mobility utilization has remained stable at just under 20% in revision THA cases and represents the most commonly used bearing surface in revision performed for instability (65%). Notably, reporting of this metric differs between the 2024 and prior reports compared with the current report, likely due to misclassification of some DM constructs as smaller diameter femoral heads (ie, ≤ 28 mm). For example, DM use in revision THA for instability was reported as 34.3% in 2023 in the 2024 Annual Report, compared with 64.6% in the 2025 Annual Report. Larger femoral heads (≥36 mm) are the predominant head size in revision THA, with a continued increase in the use of heads ≥40 mm.

### Trends in primary TKA

• For the first time since the inception of the AJRR, medial congruent inserts became the most commonly utilized insert design in 2024, increasing to 43% from 33% in 2023 and surpassing posterior-stabilized designs, which declined to 34% in 2024 from 40% in 2023. Notably, the use of cruciate retaining liners continues to decline to 6.6% compared to 35.6% in 2012.
• The use of HXPE (47% in 2024 vs 46% in 2023) and antioxidant HXPE (41% in 2024 vs 39% in 2023) continues to increase, and conventional polyethylene (CPE) (12% in 2024 vs 15% in 2023) continues to decline in primary TKA. Similar to the 2024 Annual Report, there is no significant difference in cumulative revision rates when comparing HXPE or antioxidant HXPE compared to CPE in men or women older than 65 years of age beyond 8 years of follow-up.
• Although patellar resurfacing remains the most common technique among TKA surgeons, its utilization continues to decline. In 2024, patellar resurfacing was performed in 86% of primary TKAs, compared to 87% in 2023 and 89% in 2022. This trend began in 2012, when 96% of patellae were resurfaced. Despite this decline, resurfacing rates in the US remain higher than those reported in international registries, including the United Kingdom (51.7%), Australia (78.1%), Canada (50.3%), and New Zealand (55.6%) [20]. At 10 years of follow-up, there is no difference in cumulative survival among Medicare patients aged 65 years and older for primary TKA with or without patellar resurfacing (adjusted HR 1.0; 95% CI 0.9-1.1; *P* = .90).
• While cement remains the predominant mode of fixation in primary TKA, the use of cementless fixation remains on the rise increasing to 22% in 2024 up from 21% in 2023. Over the past decade, cementless utilization has grown nearly 10-fold, from just 2% in 2012. Compared with the 2024 Annual Report, cementless primary TKA demonstrated significantly higher early revision rates in patients ≥ 65 years (females: 1-18 months, adjusted HR 1.24, 95% CI 1.12-1.38, *P* < .01; males: 3-12 months, adjusted HR 1.18, 95% CI 1.03-1.35, *P* < .05), and significantly lower mid-term revision rates in males ≥65 years (30-72 months, adjusted HR 0.68, 95% CI 0.54-0.85, *P* < .01), with no significant differences outside these time intervals. In patients <65 years, cementless fixation was associated with significantly lower mid-term (2-7.5 years) revision rates in males (adjusted HR 0.65, 95% CI 0.56-0.75, *P* < .001), while females had significantly higher early (0-6 months) revision rates with cementless (adjusted HR 1.23, 95% CI 1.1-1.4, *P* < .01) and hybrid fixation (adjusted HR 1.45, 95% CI 1.1-1.89, *P* < .01), with no differences outside these time intervals.

### Trends in unicompartmental knee arthroplasty

• Over the past 5 years, unicompartmental knee arthroplasty (UKA) has accounted for approximately 4% of all knee arthroplasty procedures, which is similar to the percentage of UKAs performed in 2024 (4%).
• The cumulative revision rates of UKA compared to primary TKA are significantly higher after 3 months (adjusted HR 1.4, 95% CI 1.3-1.4; *P* < .01) and beyond 6 years (adjusted HR 1.9, 95% CI 1.6-2.3, *P* < .01).

### Trends in revision TKA

• Infection and inflammatory reactions remain the leading cause for overall revision, increasing by 15% to account for 52% of all revision TKA procedures, up from 45% in 2023. This has steadily increased over time from 31% in 2012. Aseptic loosening remains the second most common reason for knee revision at 29%.
• Infection was also the most frequent reason for re-revision TKA at 40%, up from 37% in 2023.

Each year, the AJRR Annual Report continues to provide comprehensive data and in-depth analysis on hip and knee arthroplasty practice trends among surgeons in the United States, including implant survivorship and utilization in an effort to advance musculoskeletal care. Future strategic directives, including a partnership with Epic to enable more accurate, real-time data capture and the development of infrastructure to support nested randomized trials, will further strengthen the AJRR and establish it as one of the world’s leading orthopaedic registries.

## CRediT authorship contribution statement

**David Novikov:** Writing – review & editing, Writing – original draft, Data curation. **Niall Cochrane:** Writing – review & editing, Writing – original draft. **James I. Huddleston:** Writing – review & editing, Supervision. **Brett R. Levine:** Writing – review & editing, Supervision. **Anna Cohen-Rosenblum:** Writing – review & editing.

## Conflicts of interest

Brett Levine receives royalties from Link; is a paid consultant for Link, Zimmer-Biomet, Enovis; Royalties, receives financial or material support from Slack, Elsevier, Human Kinetics, Wolters-Kluwer; serves on the editorial/governing board for JOA, Orthopaedics, AT (EIC); and serves as a board member for AAOS—EQBV, HS/KS Digital Media Committee. David Novikov serves as a board member for AAHKS. Niall Cochrane serves as a board member for AAHKS. James I. Huddleston III receives royalties from Depuy, ZimmerBiomet, Exactech; is a Paid consultant for Depuy, ZimmerBiomet, Exactech; holds stock or stock options in Corin; receives royalties, financial or material support from WoltersKluwer; serves on the editorial/governing board for JOA; and serves as a board member for AAOS, AJRR, AAHKS, Knee Society, Hip Society. All other authors declare no potential conflicts of interest.

For full disclosure statements refer to https://doi.org/10.1016/j.artd.2026.102121.

## Appendix A Supplementary data

Conflict of Interest Statement for Levine

Conflict of Interest Statement for Novikov

Conflict of Interest Statement for Cochrane

Conflict of Interest Statement for Cohen-Rosenblum

Conflict of Interest Statement for Huddleston
