# Supplementary material for: Highlights of the 2025 American Joint Replacement Registry Annual Report
Source: Arthroplast Today. 2026 Aug 22;41:102121. doi: 10.1016/j.artd.2026.102121 (PMC13524717; doi:10.1016/j.artd.2026.102121)
Supplement: Supplementary file 1 [file mmc4.pdf]

CONFLICT OF INTEREST STATEMENT

American Association of Hip and Knee Surgeons

(Adopted from the American Academy of Orthopaedic Surgeons disclosure statement)

The following form **must be filled out completely and submitted by each author (example, 6 authors, 6 forms). All items require a response. If there is no relevant disclosure for a given item, enter "*None*."**

Highlights of the 2025 American Joint Replacement Registry Annual Report

Manuscript Title

1. Royalties from a company or supplier (The following conflicts were disclosed)
none

2. Speakers bureau/paid presentations for a company or supplier (The following conflicts were disclosed)
none

3A. Paid employee for a company or supplier (The following conflicts were disclosed)
none

3B. Paid consultant for a company or supplier (The following conflicts were disclosed)

Smith and Nephew, ZB, Heraeus

3C. Unpaid consultants for a company or supplier (The following conflicts were disclosed)

none

4. Stock or stock options in a company or supplier (The following conflicts were disclosed)

none

5. Research support from a company or supplier as a Principal Investigator (The following conflicts were disclosed)

none

6. Other financial or material support from a company or supplier (The following conflicts were disclosed)
none

7. Royalties, financial or material support from publishers (The following conflicts were disclosed)
Elseiver, JBJS

8. Medical/Orthopaedic publications editorial/governing board (The following conflicts were disclosed)

Arthroplasty Today, Journal of Arthroplasty

9. Board member/committee appointments for a society (The following conflicts were disclosed)
AAOS, SICOT, RJOS

Each author must sign AND print or type his/her name, date and submit a separate form

In addition, one BLINDED Conflict of Interest form (no author names used) should be submitted per manuscript with all author disclosures.

Anna Cohen-Rosenblum	[signature]	4/27/26
Author Name (Print or Type)	Author Signature	Date
