# Supplementary material for: Highlights of the 2025 American Joint Replacement Registry Annual Report
Source: Arthroplast Today. 2026 Aug 22;41:102121. doi: 10.1016/j.artd.2026.102121 (PMC13524717; doi:10.1016/j.artd.2026.102121)
Supplement: Supplementary file 2 [file mmc1.docx]

# CONFLICT OF INTEREST STATEMENT

***American Association of Hip and Knee Surgeons***

(Adopted from the American Academy of Orthopaedic Surgeons disclosure statement)

The following form **must be filled out completely and submitted by each author (example, 6 authors, 6 forms).**

**All items require a response. If there is no relevant disclosure for a given item, enter "*None*.”**

Highlights of the 2025 American Joint Replacement Registry Annual Report

Manuscript Title

1.	Royalties from a company or supplier (The following conflicts were disclosed)

Link

2.	Speakers bureau/paid presentations for a company or supplier (The following conflicts were disclosed)

None

3A.	Paid employee for a company or supplier (The following conflicts were disclosed)

None

3B.	Paid consultant for a company or supplier (The following conflicts were disclosed)

Link, Zimmer-Biomet, Enovis

3C.	Unpaid consultants for a company or supplier (The following conflicts were disclosed)

None

4.	Stock or stock options in a company or supplier (The following conflicts were disclosed)

None

5.	Research support from a company or supplier as a Principal Investigator (The following conflicts were disclosed)

None

6.	Other financial or material support from a company or supplier (The following conflicts were disclosed)

None

7.	Royalties, financial or material support from publishers (The following conflicts were disclosed)

Slack, Elsevier, Human Kinetics, Wolters-Kluwer

8.	Medical/Orthopaedic publications editorial/governing board (The following conflicts were disclosed)

JOA, Orthopedics, AT (EIC)

9.	Board member/committee appointments for a society (The following conflicts were disclosed)

AAOS—EQBV, HS/KS Digital Media Committee

**Each author must sign AND print or type his/her name, date and submit a separate form**

In addition, one BLINDED Conflict of Interest form (no author names used) should be submitted per manuscript with all author disclosures.

Brett Levine	04/23/2026

Author Name (Print or Type)	Author Signature	Date
