# Supplementary material for: Highlights of the 2025 American Joint Replacement Registry Annual Report
Source: Arthroplast Today. 2026 Aug 22;41:102121. doi: 10.1016/j.artd.2026.102121 (PMC13524717; doi:10.1016/j.artd.2026.102121)
Supplement: Supplementary file 5 [file mmc5.docx]

# INDIVIDUAL CONFLICT OF INTEREST STATEMENT

***American Association of Hip and Knee Surgeons***

(Adopted from the American Academy of Orthopaedic Surgeons disclosure statement)

The following form **must be filled out completely and submitted by each author (example, 6 authors, 6 forms).**

**All items require a response. If there is no relevant disclosure for a given item, enter "*None*.”**

Highlights of the 2025 American Joint Replacement Registry Annual Report

**Manuscript Title**

1.	Royalties from a company or supplier (The following conflicts were disclosed) Depuy, ZimmerBiomet, Exactech

:

2.	Speakers bureau/paid presentations for a company or supplier (The following conflicts were disclosed) none

:

3A.	Paid employee for a company or supplier (The following conflicts were disclosed)	none

:

3B.	Paid consultant for a company or supplier (The following conflicts were disclosed) Depuy, ZimmerBiomet, Exactech

:

3C.	Unpaid consultants for a company or supplier (The following conflicts were disclosed) none

4.	Stock or stock options in a company or supplier (The following conflicts were disclosed)	Corin

5.	Research support from a company or supplier as a Principal Investigator (The following conflicts were disclosed)

: none

6.	Other financial or material support from a company or supplier (The following conflicts were disclosed)	none

:

7.	Royalties, financial or material support from publishers (The following conflicts were disclosed) WoltersKluwer

:

8.	Medical/Orthopaedic publications editorial/governing board (The following conflicts were disclosed) JOA

9.	Board member/committee appointments for a society (The following conflicts were disclosed) AAOS, AJRR, AAHKS, Knee Society, Hip Society

**Each author must sign AND print or type his/her name, date and submit a separate form**

In addition, one BLINDED Conflict of Interest form (no author names used) should be submitted per manuscript with all author disclosures.

James I. Huddleston III 4-24-26

Author Name (Print or Type)	Author Signature	Date
